# The 4th NextGen Therapies for SJIA and MAS: part 1 the elephant in the room: diagnostic/classification criteria for systemic juvenile idiopathic arthritis and adult-onset still’s disease

**DOI:** 10.1186/s12969-023-00864-1

**Published:** 2024-01-05

**Authors:** Peter A. Nigrovic, Fabrizio de Benedetti, Yukiko Kimura, Daniel J. Lovell, Sebastiaan J. Vastert

**Affiliations:** 1 Division of Immunology, Boston Children’s Hospital, Karp 10210, One Blackfan Circle, Boston, MA 02115 USA; 2 Division of Rheumatology, Inflammation, and Immunity, Brigham and Women’s Hospital, Boston, MA USA; 3 Division of Rheumatology, Ospedale Pediatrico Bambino Gesù, Rome, Italy; 4 Division of Pediatric Rheumatology, Joseph M. Sanzari Children’s Hospital, Hackensack Meridian School of Medicine, Hackensack, NJ USA; 5 Division of Rheumatology, Cincinnati Children’s Hospital Medical Center, University of Cincinnati, Cincinnati, OH USA; 6 Department of Pediatric Rheumatology & Immunology and Center for Translational Immunology, University Medical Center Utrecht, Utrecht, the Netherlands

**Keywords:** SJIA, Still’s Disease, AOSD, SJIA diagnostic criteria

## Abstract

Currently, the criteria used to classify patients with SJIA are different from those used for AOSD. However, it has been recognized that the existing terms are too narrow, subdividing the Still’s population unnecessarily between pediatric-onset and adult-onset disease and excluding an appreciable group of children in whom overt arthritis is delayed or absent. Government regulators and insurers rely upon the guidance of subject experts to provide disease definitions, and when these definitions are flawed, to provide new and better ones. The classification session at the NextGen 2022 conference helped to serve this purpose, establishing the need for a revised definitional system that transcends the fault lines that remain in existing definitions.

## Introduction

A distinct childhood disease characterized by fever, rash, systemic inflammation, and arthritis was first described by George Frederic Still in 1897 and incorporated formally as a subtype of childhood arthritis in 1977 [1, 2]. Since that time, this condition has been referred to by the eponym *Still’s disease* or the descriptive names *systemic-onset juvenile rheumatoid arthritis*, *systemic-onset juvenile chronic arthritis*, and most recently the International League of Associations for Rheumatology (ILAR) term *systemic juvenile idiopathic arthritis* (SJIA) [3]. Individuals aged 16 years and older with a related presentation are described as having *adult-onset Still’s disease* (AOSD).

While clinical practice tolerates a flexible application of these terms, research requires rigor, rendering the fine details of nomenclature important. Different terminologies delineate distinct sets of patients who can and cannot be enrolled in a particular study, and therefore to whom resulting observations can be considered to apply. Consequently, regulatory authorities and insurance companies rely on these terms with respect to drug approval decisions, determining who can and cannot access treatment. Seemingly minor differences in terminology can thereby translate directly into real-world consequences for patients and families [4]. Taking the term *Still’s disease* to include both SJIA and AOSD, the question of who does and does not have this syndrome is the elephant in the room with respect to how research can be brought to bear to guide in the care of Still’s patients who desperately need new treatment options.

## Fault lines in Still’s disease definitions

Four classification criteria sets in active use in Still’s disease are presented in Table 1. Beyond multiple small differences, two fundamental “fault lines” divide these definitions from each other: (1) the *age cutoff* below or above which a disease must have begun; and (2) whether overt *arthritis* is required.

**Table 1:** Selected criteria for systemic juvenile idiopathic arthritis and adult-onset Still’s disease

Disease	AOSD	Systemic JIA	Systemic JIA	Systemic JIA
Lead author or sponsor	**Yamaguchi**	**ILAR**	**CARRA**	**PRINTO**
Publication date	1992	2004	2012	2019
Inclusion criteria	5 criteria, including at least 2 major criteria Major criteria • fever ≥ 39C ≥ 1 week • arthralgia ≥ 2 weeks • typical rash • WBC ≥ 10 k including ≥ 80% granulocytes Minor criteria • sore throat • lymphadenopathy and/or splenomegaly • liver dysfunction • negative RF and ANA	Arthritis of unknown etiology beginning before the 16^th^ birthday and persisting ≥ 6 weeks Fever ≥ 2 weeks, daily ≥ 3d Plus one or more of • evanescent erythematous rash • generalized lymphadenopathy • hepatomegaly and/or splenomegaly • serositis	Arthritis beginning between age 6 months and 18y of age (before 19^th^ birthday) and persisting ≥ 1 week Fever ≥ 2 weeks, must exhibit quotidian pattern at some point Plus one or more of • evanescent erythematous rash • generalized lymphadenopathy • hepatomegaly or splenomegaly • pericarditis, pleuritis and/or peritonitis	Onset before 18^th^ birthday Fever ≥ 2 weeks, daily ≥ 3d + 2 major criteria or 1 major / 2 minor criteria Major criteria • evanescent erythematous rash • arthritis (any duration) Minor criteria • generalized lymphadenopathy, hepato- and/or splenomegaly • serositis • arthralgia ≥ 2wk • WBC ≥ 15 k with neutrophilia
Exclusion criteria	Infections (especially sepsis and mononucleosis), malignancy (especially lymphoma), rheumatic disease (especially vasculitis)	HLA-B27 + and onset ≥ age 6y, RF twice ≥ 3 months apart, personal or 1^st^ degree family history of psoriasis, ankylosing spondylitis, enthesitis related arthritis, sacroiliitis with inflammatory bowel disease, Reiter’s, acute anterior uveitis	infection, malignancy, or other causes that can present with similar symptoms to systemic JIA	other known conditions
Notes	Age of onset not specified		Arthritis must be observed by a physician; duration ≥ 1 week specified in ref. (24)	PRINTO definition of JIA requires inflammatory disease to persist ≥ 6 weeks

*Abbreviations*: *ANA* Antinuclear antibody, *AOSD* Adult-onset Still’s disease, *CARRA* Childhood Arthritis and Rheumatology Research Alliance, *JIA* Juvenile idiopathic arthritis, *PRINTO* Paediatric Rheumatology International Trials Organization, *RF* Rheumatoid factor, *WBC* White blood cell count, *y* year

References: Yamaguchi [5], ILAR [3], CARRA [6, 7], PRINTO [8]

### Age cutoff

The categorical separation of pediatric-onset and adult-onset disease is a hallmark feature of all current arthritis nomenclatures [9]. Historically, juvenile arthritis begins before the 16^th^ birthday, an age cutoff that first appeared – explicitly as a matter of arbitrary convenience, rather than biology – in one of the earliest series describing arthritis in children [10–12]. That the same cutoff has been applied to divide SJIA from AOSD is especially remarkable because the original description of AOSD in 1971 was by an expert in pediatric rheumatology who sought to report adult cases of the same syndrome observed in childhood [13]. The recent CARRA and PRINTO definitions extend the age spectrum to the 19^th^ and 18^th^ birthday respectively, but still retain the categorical divide between SJIA and AOSD (Table 1) [6, 8].

Observational series suggest that SJIA and AOSD do indeed differ in a few clinical features, specifically the likelihood of long standing chronic arthritis (a so-called “persistent” course, ~ 30% in AOSD vs. ~ 50% in SJIA) and the presence of sore throat (50–90% in AOSD series, typically not enumerated in SJIA series) (Table 2). A recent report sought to characterize the phenotype of pediatric and adult Still’s disease in parallel, confirming substantial similarity [14]. Of note, different diagnostic criteria were employed for pediatric and adult patients (ILAR for SJIA and Yamaguchi for AOSD, respectively), and all data collection was retrospective, such that some remaining differences may have been definitional (for example, all pediatric patients but only 60% of adults developed overt arthritis) or due to variation in ascertainment. For example, pediatric rheumatologists do not routinely query SJIA patients about sore throat, not least because many children with SJIA are too young to report this symptom reliably.

**Table 2:** Comparison of clinical features in pediatric and adult Still’s disease from representative series

Series	Schneider	Mozziconacci and Prieur	Russo	Pouchot	Gerfaud-Valentin	Ichida	Ruscitti	Ruscitti	
Date	1992	2003/2004	2013	1991	2014	2014	2016	2022	
Terminology	soJRA	soJRA	sJIA	ASD	AOSD	AOSD	AOSD	sJIA	AOSD
n	38	100	132	62	57	71	100	166	194
Age of onset (y)	6.3	4.5	5	24	36	32	45	5	41
female	55	51	59	45	53	65	66	52	47
Fever	100	100	100	100	95	99	100	100	98
Rash	90	90	71	87	77	79	78	73	73
Arthritis	90	80	100	94	95^a^	93	86^a^	100	60
Sore throat	NR	NR	NR	92	53	57	64	10	59
Persistent course	53	50	58	36	26	NR	33	22	14

*Abbreviations*: *AOSD* Adult-onset Still’s disease, *ASD* Adult Still’s disease, *soJRA* Systemic-onset juvenile rheumatoid arthritis, *sJIA* Systemic juvenile idiopathic arthritis. Age of onset is mean or median, depending on series

^a^Arthralgia or arthritis in 95%. References: Schneider [15], Mozziconacci and Prieur [16, 17], Russo [18], Pouchot [19], Gerfaud-Valentin [20], Ichida [21], Ruscitti 1996 [22], and Ruscitti 2022 [14]

Notwithstanding minor differences between SJIA and AOSD, this variation pales in comparison to the variation between individual Still’s patients, for example between younger and older children with SJIA [18]. Taking a larger view, the clinical and biological similarities between SJIA and AOSD far outnumber any differences. These include clinical presentation (Table 2), laboratory features such as IL-18 elevation, ferritin, NK cell dysfunction, and therapeutic response to IL-1 and IL-6 blockade in many patients [21, 23–27]. Accordingly, most experts regard Still’s disease as a single disease spectrum spanning pediatric and adult age groups [27–30].

### Arthritis requirement

As a form of JIA, SJIA requires by definition that patients satisfy the same general “entry criteria” as other subforms: age of onset before the 16^th^ birthday, arthritis lasting at least 6 weeks, and unknown cause [3]. A requirement for an overt inflammatory arthritis (lasting at least 1 week) remains in the CARRA modified definition of SJIA; the recently proposed PRINTO definition does not require arthritis, but accepts it as one of two major criteria, conceptually moving towards the Yamaguchi criteria for AOSD in which arthritis can be fully absent and where instead arthralgia represents a minor criterion (Table 1) [5–8].

The main benefit to including arthritis in the definition of SJIA is enhanced diagnostic certainty, since fever and systemic inflammation are observed in many conditions, including key SJIA mimics such as infection and malignancy. By contrast, it has long been recognized that overt arthritis commonly lags behind fever and other systemic manifestations of SJIA, and in some patients arthritis never appears at all, potentially as a reflection of effective therapy. Exemplifying this principle, SJIA series that pre-date the 2004 ILAR definition observed arthritis in only 80–90% of patients, roughly comparable to the frequency reported in AOSD (Table 2). In the largest direct comparison of ILAR-defined SJIA with patients considered by experts to have SJIA without arthritis, no differences were observed in clinical or laboratory features, or in therapeutic response to IL-1 blockade [31].

## Summary of presentations

Dr. Peter Nigrovic (Boston) introduced current definitions of SJIA and AOSD and focused the discussion on the definitional “fault lines” of age of onset and the presence of arthritis. He detailed the historical roots of these fault lines within Still’s disease. He showed how these divisions arose through process and custom rather than scientific rationale, and using published data, he outlined the case for a more inclusive definition of the Still’s spectrum that encompasses children and adults as well as patients with and without arthritis.

Dr. Yukiko Kimura (Hackensack) provided for consideration unpublished data on 844 SJIA patients from the CARRA Registry, of whom 10.8% did not have arthritis at their baseline visit and 8% never had arthritis during their disease course. These data indicate that, even though ILAR definitions nominally remain the gold standard, in practice pediatric rheumatologists are willing to diagnose SJIA in the absence of arthritis.

Dr. Sebastiaan Vastert (Utrecht) provided an updated review of data from the Utrecht series that has enrolled patients prospectively since 2008 [31–33]. He expanded on the comparison between Still’s patients with and without overt arthritis, showing data comparing 30 patients fulfilling ILR criteria for SJIA with 12 patients fulfilling criteria but lacking arthritis, showing that both groups were equivalent in demographic and clinical features [31]. In an expanded dataset on Dutch SJIA patients prospectively recruited with (*n* = 44) and without (*n* = 22) arthritis, patients without arthritis trended non-significantly higher in laboratory features associated with MAS, including sIL-2R, but with comparable IL-18 levels at onset. Among these 66 patients with clinical outcomes data at 12 months, complete response to anakinra was observed in 29/44 patients (66%) fulfilling ILAR SJIA criteria and 18/22 (82%) of patients clinically diagnosed with SJIA but lacking arthritis. In this larger dataset, levels of circulating IL-18 at diagnosis did not distinguish those with and without arthritis.

Dr. Daniel Lovell (Cincinnati) presented work in progress related to an ongoing clinical trial in SJIA approved by both the Food and Drug Administration and the European Medicines Agency (NCT03000439). This trial employs a further variation on the ILAR SJIA definition. Age of onset cutoff remains the 16^th^ birthday and arthritis is required at screening visit and at the baseline visit, although the duration of arthritis is not otherwise specified. The definition of SJIA is further liberalized by eliminating the family history exclusions employed in the ILAR definition, enabling patients to be enrolled even if family members have conditions such as psoriasis or ankylosing spondylitis.

Dr. Fabrizio de Benedetti (Rome) presented preliminary data from an ongoing study led by Dr. Claudia Bracaglia examining the median time from disease onset to appearance of clinical manifestations in *n* = 11 patients with SJIA (ILAR criteria) at the Ospedale Pediatrico Bambino Gesù in whom it was possible to track symptoms from onset. The earliest symptoms were fever and arthralgias, both typically present at disease onset. Rash and sore throat followed, often within days, followed by laboratory and physical exam findings such as leukocytosis and organomegaly. Arthritis lagged most among findings studied, appearing a median of 29 days from the first appearance of disease symptoms (range 2–1802 days). Comparing classification criteria for SJIA, the original ILAR criteria exhibited a sensitivity of only 36%, compared with 45% for Yamaguchi AOSD criteria, 91% for both CARRA-modified ILAR and PRINTO-modified ILAR criteria, and 100% for the ILAR criteria modified to require only one day of arthritis. Median time to achieve each criteria set (among those who ultimately met criteria) was 209 days (ILAR), 14 days (Yamaguchi), 29 days (CARRA), 20 days (PRINTO), and 29 days (ILAR modified).

## Summary of discussion

Focusing on the two fault lines, panelists and discussion participants concurred broadly that the division between SJIA and AOSD was not justified clinically or biologically, and that an approach of “lumping” rather than “splitting” would likely be most effective at assembling patients for future research collaborations. Similarly, it was broadly accepted that the presence of arthritis was not required for a secure diagnosis of Still’s disease, although heightened vigilance in such patients was recognized as essential to avoid misdiagnosis of SJIA mimics such as acute lymphoblastic leukemia. It was emphasized that this more inclusive Still’s category was expected to exhibit pathophysiologic diversity, as implied already by the preliminary distinctions between very young children and their older counterparts, good vs. poor responders to IL-1 blockade, IL-6-predominant vs. IL-18-predominant SJIA subgroups, and sJIA-like patents manifesting mutations in *LACC1/FAMIN* [18, 34–37]. Further study is required to better define which characteristics best define biologically related groups of patients within Still’s disease. A potential downside of broader grouping of patients, noted by Dr. Randy Cron (Birmingham), is that responses restricted to particular biologic subgroups could be missed; open-label studies with mechanistic follow-up investigations may be required to optimize personalized therapies.

Patient and family input was solicited. Participants noted that strict entry criteria limit the participation of patients into research, including at times some of the sickest patients. Patients who present with symptom complexes outside the mainstream may also be denied medications by insurers who adhere to narrow definitions of eligibility. Similarly, studies are required that define patient groups based on less common but more severe manifestations, such as patients who fail to respond to first-line treatment with biologics, or who break through after an initially good response, or with refractory macrophage activation syndrome or SJIA-associated lung disease.

Following the discussion, online surveys via the Zoom interface were employed to assess consensus. The following proposition was posed to physicians and then again to general participants: “We recognize that SJIA and adult-onset Still’s disease are the same disease and should not require arthritis for diagnosis, with no limit on age of onset.” Among those who voted, the formulation was approved by 92% of physicians (46 of 50) and by 97% of other participants (23 of 24).

## Summary and future directions

The criteria employed to classify patients with SJIA and AOSD have served usefully to focus research and enable the approval of new biologic agents for patients suffering from this clinical syndrome. However, increasingly there are persuasive evidence that the existing terms are too narrow, subdividing the Still’s population unnecessarily between pediatric-onset and adult-onset disease and excluding an appreciable group of children in whom overt arthritis is delayed or absent. Government regulators and insurers rely upon the guidance of subject experts to provide disease definitions, and when these definitions are flawed, to provide new and better ones. The classification session at the NextGen 2022 conference helps to serve this purpose, establishing the need for a revised definitional system that transcends the fault lines that remain in existing definitions. A consensus process involving pediatric and adult rheumatologists, together with insights from patients and families, will be important to achieve this aim and to enable a more inclusive, and also more accurate, system of categorization.

## Data Availability

All the data discussed during the meeting have now been published and appropriately referenced at the end of the manuscript.

## Abbreviations

ILAR: International League of Associations for Rheumatology
SJIA: Systemic juvenile idiopathic arthritis
AOSD: Adult-onset Still’s disease
CARRA: Childhood Arthritis and Rheumatology Research Alliance
IL: Interleukin
PRINTO: Paediatric Rheumatology International Trials Organization
